# Inhibition of NLRP3 by Fermented Quercetin Decreases Resistin-Induced Chemoresistance to 5-Fluorouracil in Human Colorectal Cancer Cells

**DOI:** 10.3390/ph15070798

**Published:** 2022-06-27

**Authors:** Ko-Chao Lee, Kuen-Lin Wu, Chia-Kung Yen, Shun-Fu Chang, Cheng-Nan Chen, Ying-Chen Lu

**Affiliations:** 1 Division of Colorectal Surgery, Department of Surgery, Chang Gung Memorial Hospital, Kaohsiung Medical Center, Kaohsiung 833, Taiwan; kmch4329@gmail.com (K.-C.L.); focus913@gmail.com (K.-L.W.); maple313@gmail.com (C.-K.Y.); 2 Department of Food Science, National Chiayi University, Chiayi 600, Taiwan; 3 Department of Medical Research and Development, Chang Gung Memorial Hospital Chiayi Branch, Chiayi 600, Taiwan; sfchang@cgmh.org.tw; 4 Department of Biochemical Science and Technology, National Chiayi University, Chiayi 600, Taiwan

**Keywords:** colorectal cancer, resistin, NLRP3, quercetin, *Lactobacillus plantarum*, drug resistance

## Abstract

The drug resistance of colorectal cancer (CRC) cells against 5-fluorouracil (5-FU) therapy is a major challenge to successful cancer treatment. While previous studies have proposed several 5-FU resistance mechanisms, the effects of the adipokines on cancer cells remain unclear. Thus, this study investigated the effect of resistin on 5-FU-treated CRC cell lines. The upregulation of NLRP3 can regulate the inflammatory responses in cancer cells and then enhance cancer progression. This study investigated the expression level and the function of NLRP3 on 5-FU-induced cytotoxicity in CRC cells and found that resistin-induced ERK activation and increased NLRP3 expression in CRC HCT-116 and DLD-1 cells were mediated by Toll-like receptor 4 (TLR4). The inhibition of TLR4 and ERK by pharmacological inhibitors attenuated the resistin-induced NLRP3 mRNA and protein levels. In contrast, the knockdown of NLRP3 enhanced the cytotoxic effects of 5-FU. Furthermore, quercetin is an effective chemopreventive compound. This study showed that quercetin fermented by *Lactobacillus* could exhibit low cytotoxicity on normal mucosa cells and improve the function of inhibiting CRC cells. The treatment of CRC cells with fermented quercetin increased the cytotoxicity and enhanced cell death in the presence of resistin. In this study, fermented quercetin induced the cytotoxicity and cell death of 5-FU in resistin-treated CRC cells, which is associated with the downregulation of NLRP3 expression and ERK phosphorylation. These results indicate the role of NLRP3 in the development of drug resistance to 5-FU in CRC cells. Elucidating the mechanism regarding the cytotoxicity effect of quercetin may provide another vision for the development of a chemotherapy strategy for CRC in the future.

## 1. Introduction

Colorectal cancer (CRC) is one of the most common cancer types and the second leading cause of cancer-related deaths globally. Although recent progress and emerging opportunities have been made in the treatment of CRC, which has significantly improved the clinical outcomes of patients with CRC, the recurrence and metastasis of cancer cells are still the main causes of death from this disease [1]. Obesity is associated with an increased risk of CRC, although the exact mechanism has not yet been determined [2]. Adipocytes can secrete cytokines, which are referred to as adipokines, including leptin, adiponectin, and resistin. Variations of serum adipokine levels caused by obesity are related to the progression of a variety of malignant tumors, such as breast cancer, gastric cancer, and CRC [2,3,4]. 5-fluorouracil (5-FU) combined with oxaliplatin or irinotecan is currently the first-line chemotherapy drug for CRC. However, cancer cells may eventually develop a mechanism that inactivates these chemotherapeutic agents [5]. Thus, it is necessary to study the potential prognostic factors and therapeutic targets for patients with CRC.

Resistin is a pro-inflammatory cytokine, which is mainly secreted by human macrophages and adipocytes, and plays a role in many inflammatory pathophysiological diseases, including arthritis and atherosclerosis [6]. In addition, the serum resistin levels of patients with breast cancer, CRC, and lung adenocarcinoma gradually increase in the process of tumor progression [7,8,9]. A previous study found that the serum resistin levels of CRC patients were significantly higher than that of normal subjects [4]. It has also been reported that a higher expression of resistin in tumor tissues is associated with the poor prognosis of CRC [8]. Although there is increasing evidence that resistin plays an important role in the progression of CRC, the molecular mechanism of its function on CRC chemoresistance has not been fully evaluated.

Obesity may contribute to the progression of CRC by the induction of adipokines and chronic inflammation. Evidence has accrued regarding the role of the NOD-like receptor family pyrin domain containing 3 (NLRP3) inflammasome in adipose tissue-related inflammation and the association with CRC susceptibility [10]. It has been reported that the inflammation and tumorigenesis of cancer cells can be suppressed by inhibiting the activation of NLRP3 inflammasomes [11]. While the direct effects of NLRP3 are responsible for the secretion of IL-1β and IL-18, the other effects of NLRP3 can promote cancer cells to the EMT process and metastasis [12,13]. In addition, the function of the NLRP3 inflammasome is both a crucial mediator of inflammation and a major regulator of intestinal homeostasis [14]. Abnormal activation of NLRP3 inflammasomes has recently been found in various cancer types; however, the role of NLRP3 inflammasomes in 5-FU chemoresistance of CRC cells has not been clearly elucidated.

Phytochemicals, such as phenolic compounds or flavonoids, have received extensive attention due to their pharmaceutical capacity, including health promotion and the prevention of diseases [15]. Many phytochemicals can be transformed by the intestinal microbiota to become more active ingredients; therefore, fermentation is a useful strategy to improve the therapeutic properties of bioactive compounds [16]. Quercetin, a natural dietary flavonoid found in medicinal plants and vegetables, has great pharmacological activity in inhibiting the growth and metastasis of many cancer cells [17]; however, the effect of quercetin on increased sensitivity to chemotherapy remains unclear. The daily intake of quercetin is estimated to be up to 30 mg [18], but the intestinal absorption of quercetin is highly variable, often slow, and largely incomplete. Most quercetin is unabsorbed and may be concentrated in the intestinal lumen, which is the main reason for its sparse bioavailability after consumption.

Quercetin is the major plant flavonoid widely present in plants, and *Lactobacillus* (*L*.) *plantarum* exists in the human gastrointestinal tract and is involved in the fermentation of various plant substrates. This study aimed to investigate the possible role of resistin in the cytotoxicity of 5-FU to CRC cells. The results show that resistin stimulation activated ERK phosphorylation to upregulate NLRP3 expression mediation by TLR4 in CRC cells; thus, NLRP3 induction could further decrease the sensitivity of CRC cells to 5-FU treatment. In addition, this study investigated the cytotoxicity of quercetin from a probiotic strain *Lactobacillus* (*L*.) *plantarum*) using in vitro fermentation. Quercetin fermented by *L. plantarum* markedly decreased the resistin-induced resistance development by inhibiting NLRP3 expression. Our findings provide new insights into the role and mechanism of NLRP3 upregulation in the development of the resistance of CRC cells to chemotherapeutic drugs.

## 2. Results

### 2.1. Cytotoxicity Evaluation of Quercetin on Normal Human Colon Cell Line NCM460 after L. plantarum Fermentation

**Figure 1:**
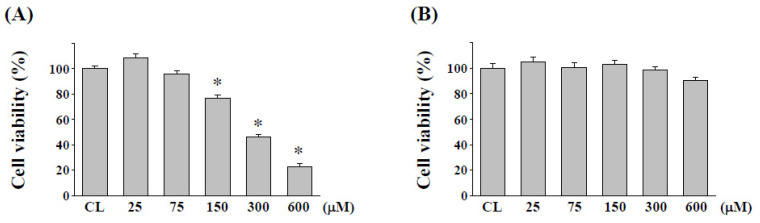
**Changes in cytotoxicity of quercetin after fermentation.** NCM460 cells were cultured in the presence of (**A**) unfermented quercetin and (**B**) fermented quercetin for 24 h, and cell cytotoxicity was determined by MTS assay. Data represent the mean ± SEM from three independent experiments; * *p* < 0.05 vs. CL NCM460 cells.

The cytotoxicity effect of quercetin before and after *L. plantarum* fermentation was determined using MTS assays with a normal human mucosa cell line NCM460. The MTS assay demonstrated that incubation with unfermented quercetin induced significant reductions in cell viability in concentrations of 150 μM or greater (Figure 1A). After *Lactobacillus*-fermentation, the cytotoxicity of quercetin was completely changed at a very high concentration without cytotoxicity up to 600 μM (Figure 1B).

### 2.2. Resistin Promotes 5-FU Resistance in CRC Cells

**Figure 2:**
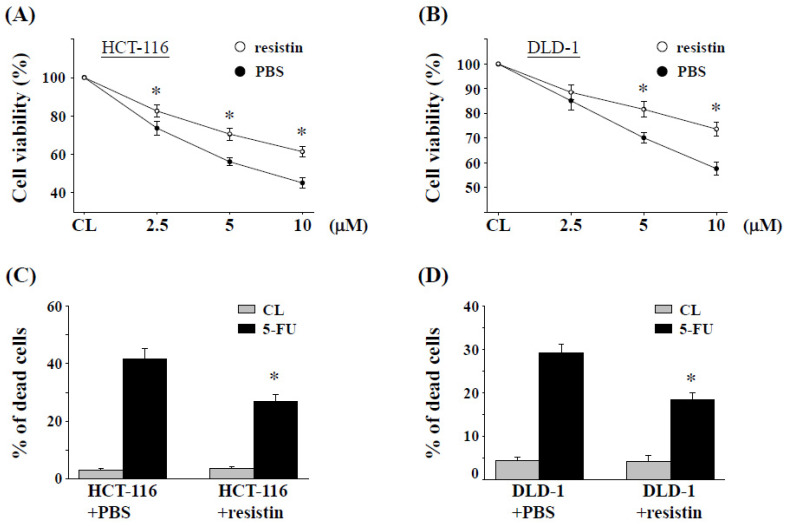
**Resistin promotes 5-FU resistance in CRC cells.** (**A**) HCT-116 cells and (**B**) DLD-1 cells pretreated with resistin (50 ng/mL) for 12 h were further treated with the indicated doses of 5-FU (0–10 μM) for 48 h in the presence of resistin, and cell viability was determined by MTS assay. Cells treated with PBS served as the control. The OD value of control cells was taken as 100% viable. (**C**) HCT-116 cells and (**D**) DLD-1 cells pretreated with 50 ng/mL resistin for 12 h were further treated with 5 μM 5-FU for 48 h in the presence of resistin, then both unattached and attached cells were collected and stained with trypan blue dye, and the number of dead cells was manually counted. The percentage of trypan blue-positive cells represented the population of dead cells. Data represent the mean ± SEM from three independent experiments; * *p* < 0.05.

It was hypothesized that increased levels of resistin might confer resistance of CRC cells against 5-FU therapy-induced cell death. To test this, CRC lines HCT-116 and DLD-1 were pretreated with resistin (50 ng/mL) or a vehicle (PBS) for 12 h, and then they were exposed to increasing doses of 5-FU (2.5–10 μM) in the presence or absence of resistin for an additional 48 h. The effect on the cytotoxicity of CRC cells was examined using an MTS assay (Figure 2A,B) and trypan blue dye exclusion assay (Figure 2C,D). Our data reveal that 5-FU treatment resulted in significant cell death in both HCT-116 (Figure 2A) and DLD-1 (Figure 2B) cells. Notably, the resistin treatment significantly protected CRC cells from 5-FU-induced cell death (Figure 2C,D).

### 2.3. Resistin Increases the NLRP3 Expression to Influence the 5-FU Cytotoxic Effect on CRC Cells

**Figure 3:**
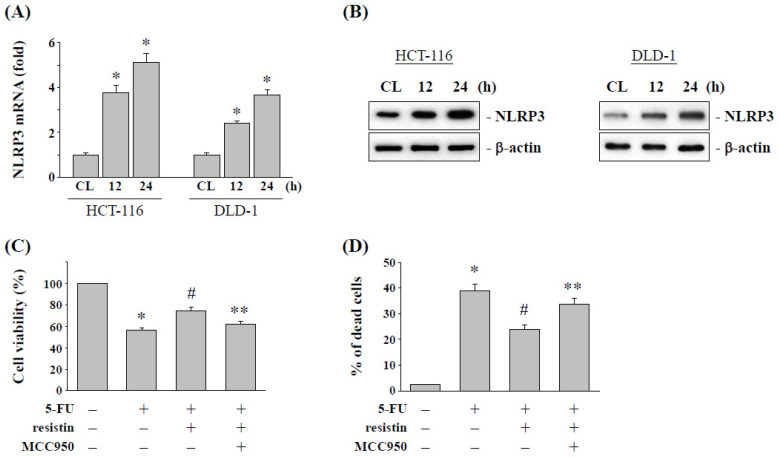
**Inhibition of NLRP3 expression in CRC cells enhances the cytotoxicity induced by 5-FU.** HCT-116 and DLD-1 cells were kept as the control (CL) or treated with resistin (50 ng/mL) for the indicated times. The mRNA (**A**) and protein (**B**) expression of NLRP3 were determined by real-time PCR and Western blotting, respectively. Data in (**A**) are shown as mean ± SEM from three independent experiments. * *p* < 0.05 versus CL. Results in (**B**) are representative of three independent experiments with similar results. (**C**,**D**) HCT-116 cells were kept as the control (CL) or treated with resistin for 12 h, then cultured in 5-FU for a further 48 h. After being treated with resistin, HCT-116 cells were pretreated with a specific inhibitor for NLRP3 (MCC950) and then treated with 5-FU (5 μM). (**C**) HCT-116 cell viability was assayed by the MTS assay. (**D**) HCT-116 cells were collected and stained with trypan blue dye, and the number of dead cells was manually counted. The percentage of trypan blue-positive cells represent the population of dead cells. Data represent the mean ± SEM from three independent experiments; * *p* < 0.05 vs. cells without treatment. ^#^ *p* < 0.05 vs. cells treated with 5-FU; ** *p* < 0.05 vs. cells treated with resistin and 5-FU.

CRC HCT-116 and DLD-1 cells were cultured with resistin for 12 and 24 h, and then the mRNA and protein expression of NLRP3 were examined. The cultivation of cells with 50 ng/mL resistin significantly induced NLRP3 mRNA (Figure 3A) and protein (Figure 3B) expression in both HCT-116 and DLD-1 cells. In order to determine the role of NLRP3 expression in the cell cytotoxicity of 5-FU induction with resistin treatment, HCT-116 cells were pretreated with NLRP3-specific inhibitor MCC950 and then incubated with or without resistin (50 ng/mL) and 5-FU (5 μM) for another 48 h. The results showed that the inhibition of NLRP3 significantly decreased the 5-FU resistance of HCT-116 cells induced by resistin, which resulted in the recovery of resistin, increased cell viability (Figure 3C), and decreased cell death (Figure 3D).

### 2.4. ERK Signaling Regulates the Resistin-Induced NLRP3 Expression and Subsequent 5-FU Resistance in HCT-116 Cells

**Figure 4:**
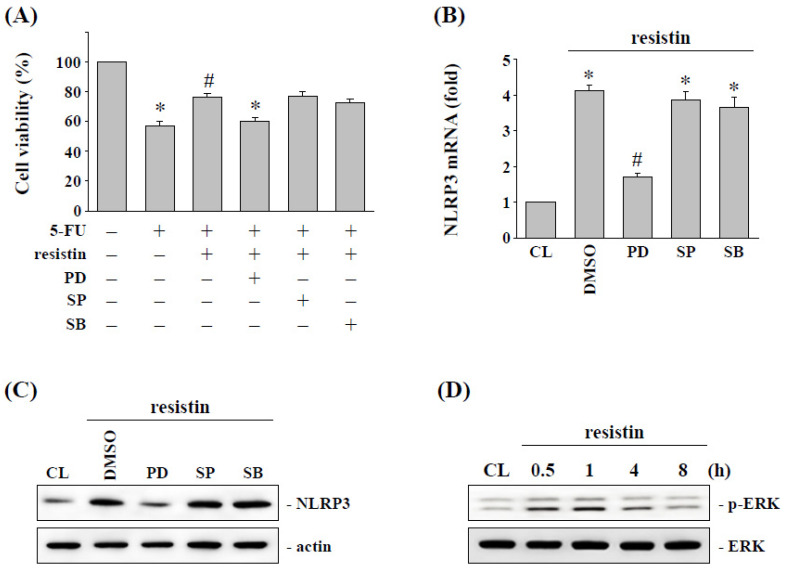
**ERK signaling regulates resistin-increased NLRP3 expression and subsequent cell cytotoxicity of HCT-116 cells.** (**A**–**C**) HCT-116 cells were kept as the control (CL) or treated with resistin for 12 h, then cultured in 5-FU for a further 48 h. After being treated with resistin, HCT-116 cells were pretreated with specific inhibitors for ERK (PD98095), JNK (SP600125), and p38 (SB203580) and then treated with 5-FU. (**A**) Cell viability was assayed using the MTS assays. (**B**) The mRNA expression of NLRP3 was determined by real-time PCR. (**C**)The protein expression of NLRP3 was determined by Western blotting. (**D**) HCT-116 cells were kept as the control (CL) or treated with resistin for the indicated times. The ERK phosphorylation was determined by Western blotting. Data in (**A,B**) are shown as mean ± SEM from three independent experiments. * *p* < 0.05 versus CL. ^#^ *p* < 0.05 versus DMSO/5-FU-treated cells. Results in (**C**,**D**) are representative of three independent experiments with similar results.

In order to determine whether resistin induced NLRP3 expression and subsequent 5-FU resistance in HCT-116 cells are regulated by MAPK signaling, HCT-116 cells were incubated with DMSO or specific inhibitors for MAPKs, and then treated with resistin (50 ng/mL). HCT-116 cells were pretreated with MAPK inhibitors and then incubated with or without resistin (50 ng/mL) and 5-FU (5 μM) for another 48 h. The results showed that the inhibition of ERK significantly decreased the 5-FU resistance of HCT-116 cells induced by resistin, which resulted in a recovery in the resistin-increased cell viability (Figure 4A). Pretreating HCT-116 cells with ERK inhibitor PD98059 significantly inhibited the resistin-increased NLRP3 mRNA (Figure 4B) and protein (Figure 4C) expression. Moreover, the cells treated with resistin (50 ng/mL) resulted in significant increases in ERK phosphorylation in HCT-116 cells (Figure 4D).

### 2.5. Toll-Like Receptor 4 (TLR4) Regulates the Resistin-Induced NLRP3 Expression and Subsequent 5-FU Resistance in HCT-116 Cells

**Figure 5:**
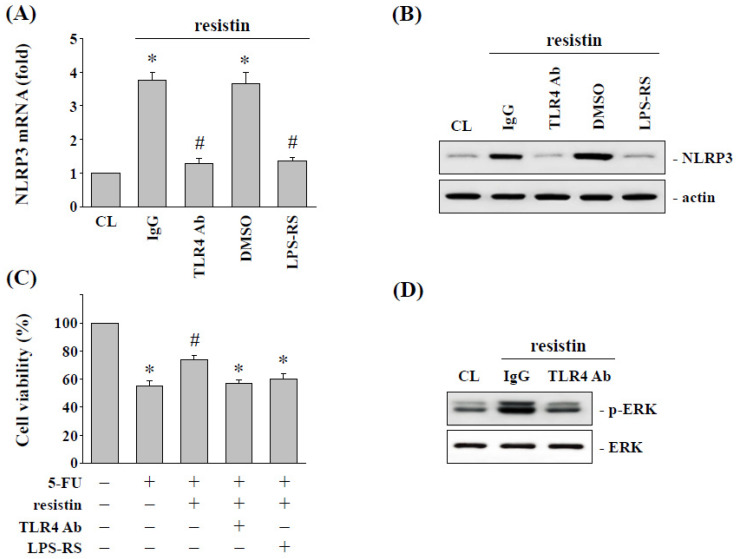
**TLR4 signaling regulates resistin-activated NLRP3 expression in HCT-116 cells.** (**A**–**C**) HCT-116 cells were kept as the control (CL) or treated with resistin for 12 h, then cultured in 5-FU for a further 48 h. After being treated with resistin, HCT-116 cells were pretreated with a neutralizing antibody for TLR4 (Ab-TLR4) or a specific inhibitor for TLR4 (LPS-RS) and then treated with 5-FU. (**A**) The mRNA expression of NLRP3 was determined by real-time PCR. (**B**) The protein expression of NLRP3 was determined by Western blotting. (**C**) Cell viability was assayed using the MTS assays. (**D**) HCT-116 cells were kept as the control (CL) or treated with resistin for 1 h. Before being treated with resistin, HCT-116 cells were pretreated with a neutralizing antibody for TLR4 (Ab-TLR4). The ERK phosphorylation was determined by Western blotting. Data in (**A**,**C**) are shown as mean ± SEM from three independent experiments. * *p* < 0.05 versus CL. ^#^ *p* < 0.05 versus DMSO/5-FU-treated cells. Results in (**B**,**D**) are representative of three independent experiments with similar results.

TLR4 has been indicated to be the receptor for resistin stimulation [19]. In order to determine whether resistin-induced NLRP3 expression and subsequent 5-FU resistance in HCT-116 cells are regulated by TLR4, HCT-116 cells were stimulated by resistin with the addition of TLR4 neutralizing antibody or inhibitor LPS-RS. Pretreating HCT-116 cells with TLR4 neutralizing antibody and LPS-RS significantly inhibited the resistin-increased NLRP3 mRNA (Figure 5A) and protein (Figure 5B) expression. HCT-116 cells were pretreated with TLR4 neutralizing antibody or inhibitor LPS-RS and then incubated with or without resistin (50 ng/mL) and 5-FU (5 μM) for another 48 h. The results show that the inhibition of TLR4 significantly decreased the 5-FU resistance of HCT-116 cells induced by resistin, which resulted in a recovery in the resistin-increased cell viability (Figure 5C). In addition, treating HCT-116 cells with TLR4 neutralizing antibodies also significantly inhibited the resistin-induced ERK phosphorylation (Figure 5D).

### 2.6. Quercetin Fermentation by L. plantarum LYC219 Enhances 5-FU-Induced Cell Cytotoxicity in HCT-116 Cells with Resistin Stimulation

**Figure 6:**
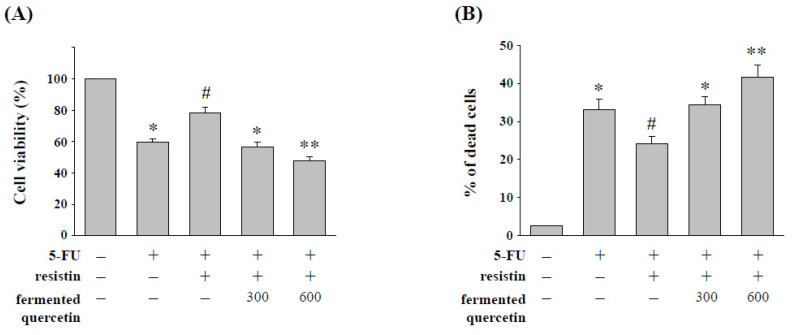
**Fermented quercetin enhances 5-FU-induced cell cytotoxicity in HCT-116 cells with resistin stimulation.** HCT-116 cells were kept as the control (CL) or treated with resistin for 12 h, then cultured in 5-FU for a further 48 h. After being treated with resistin, HCT-116 cells were treated with different doses of fermented quercetin (300–600 μM) and then treated with 5-FU. (**A**) HCT-116 cell viability was assayed by the MTS assay. (**B**) HCT-116 cells were collected and stained with trypan blue dye, and the number of dead cells was manually counted. The percentage of trypan blue-positive cells represent the population of dead cells. Data represent the mean ± SEM from three independent experiments; * *p* < 0.05 vs. cells without treatment. ^#^ *p* < 0.05 vs. cells treated with 5-FU; ** *p* < 0.01 vs. cells treated with resistin and 5-FU.

Quercetin has been reported to have great potential for use in anticancer therapy [17]. In order to determine the effect of fermented quercetin in the cell cytotoxicity of 5-FU induction with resistin treatment, HCT-116 cells were stimulated by resistin with added fermented quercetin (300 and 600 μM) and then treated with 5-FU (5 μM). The results showed that fermented quercetin significantly decreased the 5-FU resistance of HCT-116 cells induced by resistin, which resulted in a recovery in the resistin-increased cell viability (Figure 6A) and -decreased cell death (Figure 6B). However, unfermented quercetin at a noncytotoxic concentration (100 μM) did not induce the antitumor effect on resistin-treated HCT-116 cells (data not shown).

### 2.7. Fermented Quercetin Inhibits Resistin-Induced NLRP3 Expression and ERK Phosphorylation

**Figure 7:**
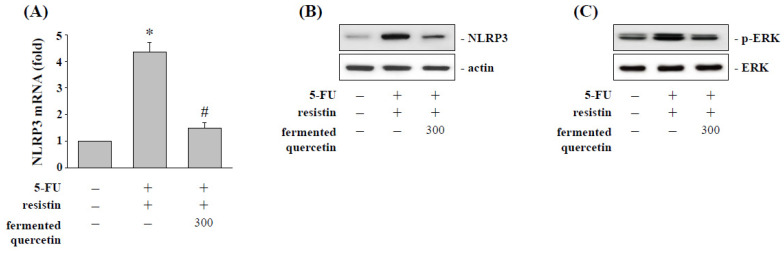
**Fermented quercetin regulates NLRP3 expression and ERK phosphorylation of resistin-treated HCT-116 cells.** HCT-116 cells were kept as the control (CL) or treated with resistin for 12 h, then cultured in 5-FU for a further 48 h. After being treated with resistin, HCT-116 cells were treated with fermented quercetin and then treated with 5-FU. The mRNA (**A**) and protein (**B**) expression of NLRP3 were determined by real-time PCR and Western blotting, respectively. (**C**) The ERK phosphorylation was determined by Western blotting. Data in (**A**) are shown as mean ± SEM from three independent experiments. * *p* < 0.05 versus CL. ^#^ *p* < 0.05 versus DMSO/5-FU-treated cells. Results in (**B**,**C**) are representative of three independent experiments with similar results.

HCT-116 cells were pretreated with fermented quercetin (300 μM) and then stimulated with resistin (50 ng/mL) for 24 h. Stimulation of HCT-116 cells with resistin induced NLRP3 mRNA (Figure 7A) and protein (Figure 7B) expression, as compared with the untreated control. However, pretreating cells with fermented quercetin significantly inhibited these inductions in resistin-stimulated HCT-116 cells (Figure 7A,B). Next, because the activities of ERK were demonstrated to regulate the NLRP3 expression under resistin stimulation in this study, this study further explored if fermented quercetin also mediates the resistin-activated ERK phosphorylation. Cells were pretreated with fermented quercetin and stimulated with resistin for 1 h. Pretreating cells with fermented quercetin significantly suppressed ERK phosphorylation as compared with the resistin-treated cells (Figure 7C).

## 3. Discussion

This study demonstrated that (i) the expression of NLRP3 in CRC cells could be induced with resistin treatments, and this upregulation could accordingly decrease the cytotoxicity of 5-FU in HCT-116 and DLD-1 cells, (ii) TLR4 and ERK signaling were involved in the NLRP3 expression and the subsequent resistin-induced decrease in cell death, and (iii) fermented quercetin plays a crucial role in the regulation of NLRP3 expression in resistin-treated CRC cells. Thus, these results explain a reversed drug resistance by which NLRP3 expression could be regulated to influence the sensitivity of CRC cells to 5-FU.

Resistin is extensively studied for its role in obesity-related inflammation and cancer progression [20]. Several studies have shown that the serum level of resistin is elevated in CRC patients, and such an increase is correlated with poor clinical outcomes in CRC [4,8,21]. A previous study showed that the resistin levels in CRC patients were significantly higher than a matched control group, and the concentration of resistin gradually increased as the tumor stage progressed [22], which indicated that resistin is a convincing biomarker for the progression of CRC malignancy. In addition, resistin is related to the survival rate, and recent studies have raised the possibility that resistin may play a role in chemotherapy. High levels of resistin are associated with less sensitivity to chemotherapy in different cancer types [23,24,25]. The development of resistance to chemotherapy agents is the main clinical challenge in CRC patients, which emphasizes the remarkable nature of such investigations. This study showed that resistin-induced NLRP3 expression, which resulted in decreased sensitivity to 5-FU-induced cytotoxicity, was a significant contributor to drug resistance in CRC cells. Moreover, the inhibition of resistin-induced NLRP3 by MCC950 or quercetin could enhance the sensitivity of anticancer drugs in CRC.

It is not yet clear whether resistin-induced NLRP3 expression will affect the 5-FU chemotherapeutic potential. It has been shown that IL-1β may weaken the efficiency of chemotherapy [26]. IL-1 receptor antagonists, in combination with chemotherapy, have synergistic antitumor effects [27]. This study showed that resistin-mediated activation of NLRP3 might decrease the chemotherapeutic potential of 5-FU. In addition, NLRP3 inflammasome might have an aggravating effect on tumor cell proliferation and metastasis. Previous studies have revealed the finding that NLRP3 is overexpressed in CRC tumor tissues [28]. Moreover, previous works have revealed that elevated expression of NLRP3 is correlated with malignant transformation and promotes drug resistance in human tumors [29]. This study demonstrated that resistin treatment could induce NLRP3 expression in human CRC HCT-116 and DLD-1 cells; on the contrary, the inhibition of NLRP3 expression could reduce the 5-FU-induced cytotoxic effect on CRC cells. Our study also revealed that NLRP3 upregulation was mediated via TLR4 and intracellular ERK signaling. ERK phosphorylation and TLR4 activation have been reported to play crucial roles in modulating drug resistance in cancer cells by contributing to the anti-apoptosis effect and promoting cell survival [30,31]. While ERK signaling has been reported to regulate the expression of NLRP3 in various cell types [32,33], a mechanism-based approach to elucidate the resistin-induced cell viability can identify how NLRP3 can contribute to cell cytotoxicity. TLR4 and ERK signaling pathways could be therapeutic targets for the development of anticancer therapies in overweight CRC patients.

Biotransformation of phytochemicals was recently demonstrated to exhibit enhanced biological function and activity after bioconversion by *Lactobacillus* fermentation [34]. Quercetin is a flavonoid compound which has outstanding anticancer potential due to its involvement in the modulation of cellular functions, inducing cell apoptosis, and inhibiting cell proliferation [17]. Quercetin has been reported to enhance the cytotoxicity and efficacy of anticancer drugs [35], inhibit the gene expression of Multidrug resistance 1, increase the activity of anticancer drugs in human uterine sarcoma cells [36], and significantly promote gemcitabine-induced cytotoxicity in pancreatic cancer cells [37]. In addition, quercetin enhanced the anticancer effect of chemotherapy in breast cancer cells through its ability to promote the loss of cell viability, apoptosis, and autophagy through the modulation of PI3K/Akt and ERK pathways [38]. The combination regimen of quercetin with other anticancer drugs may be more effective than chemotherapeutic agents as monotherapy. This study tried fermentation using our *Lactobacillus* strain LYC 219, and this *Lactobacillus*-fermentation product was enhanced for antitumor function in resistin-treated CRC cells. The inhibition of NLRP3 expression by fermented quercetin enhanced the 5-FU-induced cytotoxicity in resistin-treated CRC cells. This study investigated the possible mechanism of fermented phytochemical quercetin and its combination with 5-FU in promoting cell death in CRC cells, and the results indicate the importance of the bioconversion of natural compounds that focus on the target of the final purpose.

## 4. Materials and Methods

### 4.1. Materials

The materials for cell culture were purchased from Gibco (Grand Island, NY, USA). Protein kinase inhibitors, including MCC950 (NLRP3), PD98059 (ERK), SP600125 (JNK), SB203580 (p38), and LPS-RS (TLR4), were purchased from Sigma (St. Louis, MO, USA). Rabbit antibodies against NLRP3, phospho-ERK, ERK, and β-actin were purchased from Cell Signaling Technology (Beverly, MA, USA). The IgG and TLR4 neutralizing antibodies were purchased from Thermo (Waltham, MA, USA). All other chemicals were obtained from Sigma (St. Louis, MO, USA).

### 4.2. Fermentation of Quercetin with L. plantarum LYC219

*L. plantarum* LYC219 was obtained from the culture collection of *Lactobacillus* strains held at the Department of Food Sciences, National Chiayi University, Taiwan. Quercetin (Sigma, St. Louis, MO, USA) was prepared in 50 mM stock using DMSO and stored at −20 °C. The different working concentrations of quercetin were prepared and stored at 4 °C before use and diluted with an MRS medium. The inoculation of *L. plantarum* LYC219 was adjusted to a concentration of 1 × 10^8^ CFU/mL, and the fermentation was performed at 37 °C under anaerobic conditions by shaking at 100 rpm for 30–36 h until the late stationary phase. The fermented quercetin solutions were centrifuged at 5000× *g* rpm for 20 min, and the supernatants were collected and maintained at −20 °C before use. The equivalent amount of quercetin in the same media in the absence of inoculating bacteria was taken as the control.

### 4.3. Cell Culture

The NCM460 cells (normal human colon mucosal epithelial cell line) obtained from INCELL Corporation (San Antonio, TX, USA) were grown in M3 media supplemented with 10% FBS at 37 °C with 5% CO_2_. The CRC HCT-116 and DLD-1 cells were purchased from the cell bank of the Taiwan Food Industry Research and Development Institute (Hsinchu, Taiwan). Cells were cultured in Dulbecco’s Modified Eagle Medium supplemented with 10% FBS and 1% penicillin/streptomycin in a 37 °C, 5% CO_2_ incubator.

### 4.4. MTS Assay

An in vitro 3-(4,5-dimethylthiazol-2-yl)-5-(3-carboxymethoxyphenol)-2-(4-sulfophenyl)-2H-tetrazolium (MTS) assay was performed. Cells were cultured at 5000 per well in 96-well tissue culture plates. To assess cell viability, drugs were added after plating. At the end of the culture period, 20 μL of MTS solution (Promega, Madison, WI, USA) was added, the cells were incubated for another 2 h, and the absorbance was measured at 490 nm using an ELISA plate reader (Biorad Technologies, Hercules, CA, USA) [39].

### 4.5. Trypan Blue Dye Exclusion Assay

After treatment, 500 CRC cells were harvested, and the proportion of dead cells was determined by a hemocytometer by counting the number of cells stained with trypan blue. Trypan blue dye can be excluded from living cells but is able to penetrate dead cells. The dead cells were calculated as follows: trypan blue (+) cells ratio (%) = (stained cell number/total cell number) × 100.

### 4.6. Real-Time Quantitative PCR

The RNA was extracted from a Trizol kit and converted to cDNA by a Reverse-transcription kit. A Real-time PCR assay of the indicated genes was performed using the FastStart DNA SYBR Green I kit (Thermo, Waltham, MA, U.S.A.). The primers of the indicated genes were: NLRP3 (positive: 5′-CGTGA GTCCC ATTAA GATGG AGT-3′; negative: 5′-CCCGA CAGTG GATAT AGAAC AGA-3′) and GAPDH (positive: 5′-AGGTG AAGGT CGGAG TCAAC-3′; negative: 5′-CCATG TAGTT GAGGT CAATG AAGG-3′). The GAPDH gene was the internal control. The RT-PCR was determined in duplicate [40].

### 4.7. Western Blot Analysis

CRC cells were lysed with a lysis buffer (1% NP-40, 0.5% sodium deoxycholate, 0.1% SDS, and a protease/phosphatase inhibitor cocktail). The protein concentration was examined using the Bradford protein assay kit (Bio-Rad, Hercules, CA, USA). Equal amounts of protein samples (50 μg) were separated by SDS-polyacrylamide gel electrophoresis (PAGE) (running gel 10%, stacking gel 4%), transferred to nitrocellulose paper, and detected by adding the designated primary and horseradish peroxidase-conjugated secondary antibodies.

### 4.8. Statistical Analysis

The results are shown with mean ± standard error of the mean. Statistical analyses were measured by an independent student’s *t*-test for two groups of data, and an analysis of variance (ANOVA) was followed by Scheffe’s test for multiple comparisons. *p*-values < 0.05 were indicated as significant.

## 5. Conclusions

In summary, this study demonstrated that fermented quercetin added a cytotoxic effect with 5-FU in CRC cells through the suppression of resistin-induced NLRP3 upregulation. The results of this study suggest that the inhibition of NLRP3 might potentiate the therapeutic effect of 5-FU in patients with CRC. Thus, further study is needed to investigate the effect of fermented quercetin and other chemotherapeutic drugs, as well as their combination in obesity conditions in vivo. However, the concept of fermented quercetin combined with 5-FU seems to present a therapeutic strategy for the treatment of CRC chemoresistance.

## Data Availability

Data is contained within the article.
